# Detection and Characterization of Autoantibodies to Neuronal Cell-Surface Antigens in the Central Nervous System

**DOI:** 10.3389/fnmol.2016.00037

**Published:** 2016-05-31

**Authors:** Marleen H. van Coevorden-Hameete, Maarten J. Titulaer, Marco W. J. Schreurs, Esther de Graaff, Peter A. E. Sillevis Smitt, Casper C. Hoogenraad

**Affiliations:** ^1^Department of Biology, Division of Cell Biology, Faculty of Science, Utrecht UniversityUtrecht, Netherlands; ^2^Department of Neurology, Erasmus Medical CenterRotterdam, Netherlands; ^3^Department of Immunology, Erasmus Medical CenterRotterdam, Netherlands

**Keywords:** autoantibodies, anti-neuronal antibodies, cell-surface antigens, diagnostic testing, autoimmune encephalitis

## Abstract

Autoimmune encephalitis (AIE) is a group of disorders in which autoantibodies directed at antigens located on the plasma membrane of neurons induce severe neurological symptoms. In contrast to classical paraneoplastic disorders, AIE patients respond well to immunotherapy. The detection of neuronal surface autoantibodies in patients’ serum or CSF therefore has serious consequences for the patients’ treatment and follow-up and requires the availability of sensitive and specific diagnostic tests. This mini-review provides a guideline for both diagnostic and research laboratories that work on the detection of known surface autoantibodies and/or the identification of novel surface antigens. We discuss the strengths and pitfalls of different techniques for anti-neuronal antibody detection: (1) Immunohistochemistry (IHC) and immunofluorescence on rat/primate brain sections; (2) Immunocytochemistry (ICC) of living cultured hippocampal neurons; and (3) Cell Based Assay (CBA). In addition, we discuss the use of immunoprecipitation and mass spectrometry analysis for the detection of novel neuronal surface antigens, which is a crucial step in further disease classification and the development of novel CBAs.

## Introduction

Anti-neuronal autoimmune encephalitis (AIE) is a heterogeneous group of disorders characterized by autoantibodies that are directed at the extracellular domains of antigens in the synaptic or extra-synaptic plasma membrane. These antigens are often key players in synaptic transmission and neuronal excitability. Antibody binding to these antigens therefore directly leads to neuronal dysfunction. When the antibodies are removed, neuronal dysfunction is commonly reversed and patients often completely recover (van Coevorden-Hameete et al., 2014; Höftberger, 2015). This striking response to immunotherapy stresses the importance of early diagnosis and treatment of AIE. To achieve this, the availability of sensitive and specific tests to detect cell-surface autoantibodies is of key importance.

Whereas diagnostics in AIE is an emerging field, large experience exists with laboratory tests used for the detection of anti-neuronal antibodies in classical paraneoplastic neurological syndromes (PNS; Probst et al., 2014). Some, but not all, of the methods used in PNS diagnostics are also suitable to detect autoantibodies involved in AIE. The different requirements for diagnostic methods are mainly determined by two major differences between classical PNS antigens and AIE antigens. Classical PNS antigens are primarily located intracellular whereas AIE antigens are located in or on the plasma membrane. In addition, antibodies directed at classical PNS antigens are mostly recognizing linear epitopes, whereas surface antigens contain mostly conformational epitopes.

Core methods in PNS and AIE diagnostics are immunohistochemistry (IHC) on rat brain sections and indirect immunofluorescence (IIF) on primate cerebellum sections. In these assays all relevant antigens are present and accessible. For the detection of neuronal surface antigens also immunocytochemistry (ICC) of primary hippocampal neurons is used. Although these techniques are very useful as initial screening methods, they do not allow for the identification of the exact molecular target of the autoantibodies. In addition, these techniques require extensive experience and are labor-intensive. Therefore, for diagnostic purposes, highly specific confirmatory tests are needed. For PNS antigens immunoblotting with recombinant antigen is used most frequently as a confirmatory test (Willison et al., 2000). For surface antigens a radioimmunoassay (RIA) can be used to detect antibodies directed at a channel complex, such as voltage gated calcium channels (VGCC; Motomura et al., 1995). However, a RIA cannot discriminate between antibodies to different channel components that can be of clinical relevance, as is the case for antibodies directed to the voltage gated potassium channel (VGKC; Lai et al., 2010; Lancaster et al., 2011a; van Sonderen et al., 2016b). To test single neuronal surface antigens cell based assays (CBA) are the method of choice. In CBAs the natural environment and conformation of the antigen is mostly maintained (Willison et al., 2000).

Despite the fact that many of these techniques are currently used in AIE diagnostic and research laboratories, the methodology to detect cell-surface antibodies is not widely standardized. In this article we review the advantages and pitfalls of three different techniques for antibody detection: (1) IHC/IIF on adult rat/primate brain slices; (2) ICC on living cultured rat hippocampal neurons; and (3) CBAs for neuronal membrane proteins (for an overview see Table 1). In addition we evaluate the use of immunoprecipitation and mass spectrometry analysis for the identification of novel cell-surface antigens.

**Table 1:** **Laboratory techniques for the detection of neuronal cell-surface antibodies**.

	Number of patients reported	IHC	ICC of living neurons	CBA	Commercial CBA available	Sensitivity and specificity
**NMDAR**	>1000	Specific staining pattern hippocampus (AIE protocol) (Dalmau et al., 2007) and (Gresa-Arribas et al., 2014)	Surface labeling of excitatory synapses (Hughes et al., 2010)	Fixed (Dalmau et al., 2008) and (Gresa-Arribas et al., 2014)	Yes	*Sensitivity IHC*: CSF 100%, serum 92% (Gresa-Arribas et al., 2014) *Sensitivity fixed CBA*: CSF 100%, serum 86% (Gresa-Arribas et al., 2014) *Sensitivity IHC plus fixed CBA*: CSF 100%, serum 86% (Gresa-Arribas et al., 2014) *Specificity IHC plus fixed CBA*: CSF 100%, serum 100% (Gresa-Arribas et al., 2014)
**LGI1**	~250	Specific staining pattern of hippocampus (AIE protocol) (Lai et al., 2010)	Surface labeling of neurons, not further specified (Lai et al., 2010)	Fixed (with ADAM22/23 coexpression) (Lai et al., 2010) Fixed with added transmembrane part (van Sonderen et al., 2016c) Live (Irani et al., 2010a)	Yes	*Sensitivity IHC*: CSF 88%, serum 100% (van Sonderen et al., 2016c) *Sensitivity fixed CBA*: CSF 53%, serum 100% (van Sonderen et al., 2016c)
**Caspr2**	~100	Diffuse neuropil staining (AIE protocol) (Lancaster et al., 2011a)	Surface labeling of neurons, not further specified (Lancaster et al., 2011a)	Fixed (Lancaster et al., 2011a) Live (Irani et al., 2012)	Yes	*Sensitivity fixed CBA*: CSF 100%, serum 100% (van Sonderen et al., 2016a)
**GlyR**	~75	Neuropil of brainstem and spinal cord (PNS protocol) (Carvajal-González et al., 2014)	Not published	Live (Carvajal-González et al.^2014^)	No	*Sensitivity live CBA*: CSF: PERM 100% (Carvajal-González et al., 2014), SPS-spectrum 0% (Martinez-Hernandez et al.; 2016), serum: PERM 100% (Carvajal-González et al. 2014)*Sensitivity fixed CBA*: Serum 92–94% (disease controls) (Martinez-Hernandez et al.; 2015) (Martinez-Hernandez et al. 2016)
**GABA_B_R**	67	Diffuse neuropil staining (AIE protocol) (Lancaster et al., 2010) and (Höftberger et al., 2013)	Surface labeling of neurons, not further specified (Lancaster et al., 2010)	Fixed (Lancaster et al., 2010)	Yes	*Sensitivity fixed CBA*: CSF 100%, serum 67–93% (Lancaster et al., 2010); (Höftberger et al., 2013); and (Jeffery et al., 2013)
**DNER**	65	PC cytoplasm, punctate staining of molecular layer cerebellum (PNS protocol) (Graus et al., 1997) and (de Graaff et al., 2012)	Surface labeling of neurons overexpressing DNER (de Graaff et al., 2012)	Fixed (de Graaff et al., 2012) Live (Greene et al., 2014)	Yes	*Sensitivity fixed CBA*: Serum 100% (Probst et al., 2015)*Specificity fixed CBA*: Serum 100% (Probst et al., 2015)
**AMPAR**	64	Diffuse neuropil staining (AIE protocol) (Lai et al., 2009)	Surface labeling of excitatory synapses (Lai et al., 2009)	Fixed (Lai et al., 2009) and (Höftberger et al., 2015)	Yes	*Specificity fixed CBA*: CSF 100%, serum 70% (Höftberger et al., 2015)
**DPPX**	28	Diffuse neuropil staining (AIE protocol)	Surface labeling of both excitatory and inhibitory	Fixed (Boronat et al., 2013); (Balint et al., 2014);	Yes	Not available
		(Boronat et al., 2013); (Balint et al., 2014); and (Tobin et al., 2014)	synapses (Piepgras et al., 2015)	and (Tobin et al., 2014)
DopamineR*	26	Staining of basal ganglia (Dale et al., 2012)	Surface labeling of neurons, not further specified (Dale et al., 2012)	Live (Dale et al., 2012)	No	Not available
**mGluR1**	16	PC cytoplasm, molecular layer cerebellum (PNS protocol) (Sillevis Smitt et al., 2000) and(Lopez-Chiriboga et al., 2016)	Not published	Fixed (Lopez-Chiriboga et al., 2016) and (Iorio et al., 2013)	Yes	Not available
**IgLon5**	10	Diffuse neuropil staining (AIE protocol) (Sabater et al., 2014)	Surface labeling of neurons, not further specified (Sabater et al., 2014)	Live (Sabater et al., 2014)	No	Not available
**mGluR5**	3	Diffuse neuropil staining (AIE protocol) (Lancaster et al., 2011b)	Surface labeling of neurons, not further specified (Lancaster et al., 2011b)	Fixed (Lancaster et al., 2011b)	Yes	Not available

IHC, Immunohistochemistry; ICC, Immunocytochemistry; CBA, Cell Based Assay; PC, Purkinje cell; PERM, Progressive encephalomyelitis with rigidity and myoclonus; SPS, Stiff person syndrome; *Not confirmed by other laboratories.

## Immunohistochemistry

The reactivity of antibodies present in patient serum or CSF against rat brain proteins can be tested with IHC. In this assay 5–9 μm thick complete rat brain slices are used. Therefore all possible antigens are available and accessible, and different brain regions can be assessed. This technique has been a core method for the detection of antibodies directed at intracellular antigens in PNS. For classical PNS antigens diagnostic laboratories mostly use IIF of primate cerebellum, for which a commercial kit is available.

**Figure 1:**
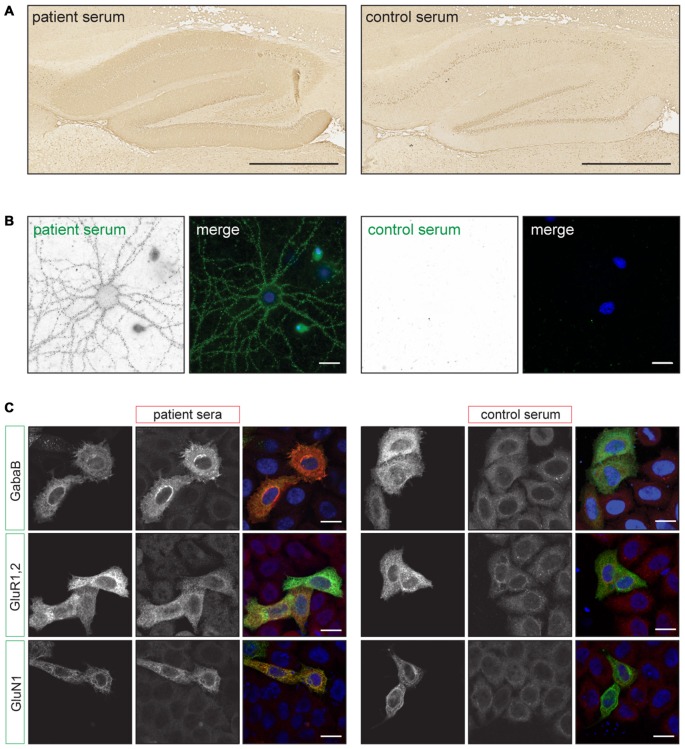
**Examples of staining patterns. (A)** Immunohistochemistry (IHC) of rat brain slices using the autoimmune encephalitis (AIE) protocol. The left picture shows neuropil staining of the hippocampus using anti-NMDA receptor (NMDAR) positive patient serum. The right picture shows staining with a healthy control serum. Scale bars: 500 μm. (**B)** Live staining of cultured rat hippocampal neurons using anti-NMDAR positive patient or healthy control serum (green). The picture shows a punctate staining pattern along the dendrites. Scale bars: 20 μm. (**C**) Examples of staining patterns of fixed Cell Based Assays (CBA) for anti-GABA_B_R, AMPAR and NMDAR antibodies. Transfected HeLa cells (green) show enhanced staining with patient serum (red) when compared to non-transfected cells and healthy control serum. Scale bars: 20 μm. Adapted from: De Bruijn and Titulaer (2016), Figure 12.1.

Although similar in many respects, the detection of synaptic surface antibodies requires a different pre-treatment of the rat brain tissue. For classical PNS antibodies brain tissue is snapfrozen, sliced with a cryostat and subsequently fixed with acetone or paraformaldehyde (PFA; Graus et al., 1997). For cell-surface antigens rat brains are fixed with PFA for 1 h at 4°C, cryoprotected in 40% sucrose for at least 24 h, snapfrozen in isopentane and subsequently sliced with a cryostat (Ances et al., 2005; Dalmau et al., 2007). By using this method of tissue preparation, antigens are well preserved and no antigen retrieval methods need to be used to obtain robust staining. The slices are incubated with serum or CSF and bound antibodies can be visualized with diaminobenzidine (DAB) peroxidase or fluorescently labeled secondary antibodies. For classical PNS antibodies, generally primate cerebellum is scored for specific staining patterns such as the distinct punctate anti-Tr/delta/notch-like epidermal growth factor-related receptor (DNER) pattern (Graus et al., 1997) or nuclear staining of anti-Hu antibodies (Sillevis Smitt et al., 2002). For cell-surface antigens, the hippocampus is scored for staining of the synapses containing gray matter, termed neuropil (Figure 1A). This neuropil staining is less robust when using the classical PNS pre-treatment of rat brain tissue. The different cell-surface antibodies can produce highly characteristic staining patterns on rat hippocampus (Dalmau et al., 2008; Montojo et al., 2015). With good quality IHC and experienced observers the recognition of these specific staining patterns may already lead to diagnosis.

Most synaptic proteins are highly homologous (for example rat-human homology on protein level for the NR1 subunit of the NMDA receptor (NMDAR) is 99.3% (source: NCBI). However, when screening for novel neuronal antigens it is important to keep in mind that due to interspecies differences some epitopes might be absent in rodents and are therefore missed on IHC.

## Immunocytochemistry on Living Primary Hippocampal Neurons

To assess antibody reactivity to extracellular epitopes live staining of cultured primary rat hippocampal neurons with patients’ serum or CSF can be performed. In theory, this way only relevant extracellular epitopes will be detected, diminishing the background signal. Also, the effects of the protein crosslinking fixative PFA, on the antigenicity of the receptor will be circumvented (Peränen et al., 1993).

For this assay it is important to use neurons that are more than 14 days in culture and have developed axons and synapses. Serum or CSF is applied when the neurons are alive and are incubated for 30–45 min at 37°C. The secondary antibody can be applied on living neurons or after fixation under non-permeabilizing conditions (Hughes et al., 2010). In both cases only extracellular epitopes will be detected and result in a punctate staining pattern along the neurites (Figure 1B). In contrast with IHC, the staining patterns of antibodies directed at different surface antigens (e.g., NMDAR, GABA_B_R) are indistinguishable (Dalmau et al., 2008; Lancaster et al., 2010). More information on the specific subcellular localization of the antigen could be determined by using co-staining with (excitatory or inhibitory) synaptic markers. However, it needs to be noted that binding of the patients antibodies to the antigen can alter the protein’s localization. For the NMDA- and AMPAR it has been shown that receptors move out of the synaptic area and become internalized (Hughes et al., 2010; Mikasova et al., 2012; Moscato et al., 2014; Peng et al., 2015). Therefore after surface labeling such co-localization studies should be interpreted with caution.

## Cell Based Assays

In a CBA a recombinant antigen is expressed by mammalian cells. The transfected cells are stained with patient serum or CSF using IIF. When the patient sample contains antibodies directed at this specific antigen, staining of the transfected cells is enhanced when compared to non-transfected cells (Figure 1C). In CBAs the antigen mostly maintains its tertiary structure and the appropriate post-translational modifications. This allows for the detection of antibodies directed at conformational epitopes.

Commercial CBAs, as well as most research laboratories, make use of human embryonic kidney (HEK) cells. HEK cells are a cell line suitable for membrane protein expression (Chaudhary et al., 2011). However, also HeLa or Chinese Hamster Ovary cells can be used as these cell lines attach more tightly to the culture plates than HEK cells and therefore wash off less easily during the staining procedure.

Controversy exists on the timing of fixation during the immunofluorescent staining procedure. With respect to the anti-NR1 CBA, some laboratories perform surface staining of live HEK cells with serum or CSF prior to fixation and permeabilization (Irani et al., 2010b; Ramberger et al., 2015), as in theory only relevant extracellular epitopes will be detected (as described in “Immunocytochemistry on Living Primary Hippocampal Neurons” Section). Other research groups fix and permeabilize the cells before immunostaining, as is also used in the commercial CBA. The only study to compare live vs. fixed CBA in an unselected way found a higher sensitivity of the fixed CBA (Gresa-Arribas et al., 2014), despite the theoretical expectations of the opposite. In addition, it needs to be noted that not all individual receptor subunits express equally well in cell lines. Some need co-expression of other subunits, auxiliary proteins or scaffolding molecules for proper receptor folding, assembly, ER export and surface expression (Leite et al., 2008; Irani et al., 2010b). This is especially important when performing live CBAs in which the receptors need to be located in the plasma membrane for antibodies to bind. It is therefore very well possible that the optimal choice between live vs. fixed CBA might be different for each receptor that is tested.

For both the fixed and live CBA one should also realize that the presence of a large number of ion channels in the plasma membrane for a longer period of time could lead to excitotoxicity and might require the addition of receptor blockers (e.g., ketamine for NMDAR) to the culture medium (Irani et al., 2010b; Ramberger et al., 2015)

Concerning the DNA constructs used for recombinant antigen expression in CBAs it is important to keep in mind that the addition of a molecular tag to a transmembrane protein for visualization purposes can affect protein trafficking and folding (Hughes et al., 2010). This could be overcome by using an untagged version in combination with cytoplasmic green fluorescent protein (GFP) to identify the transfected cells. However, in this case a commercial antibody to the antigen needs to be used to assess exact colocalization of antigen and patient antibody.

Mostly CBAs are scored with a subjective visual scoring system using epifluorescent microscopy. However, the staining intensity can vary considerably within one coverslip and accurate scoring depends heavily on the observers’ experience. For this reason in most studies two independent blinded investigators perform the scoring. Some laboratories use a semi-quantitative scoring system ranging from 0 to 4 with an increasing strength of fluorescence intensity (Leite et al., 2008). However the value of this type of semi-quantitative scoring has never been validated. To get an idea of the antibody titres it is more reliable to perform serial dilutions on IHC (Gresa-Arribas et al., 2014).

Alternatively, the evaluation of CBAs using fluorescence-activated cell sorting (FACS) is less dependent on experience. In addition it could provide a quantitative method for determining antibody titres without the need of testing serial dilutions. CBA with FACS based scoring has been used for the detection of anti-aquaporin-4 antibodies in neuromyelitis optica (De Vidi et al., 2011) and anti-NMDAR (Amatoury et al., 2013). However, only one study compared visual scoring of CBA with FACS based analysis. This study showed lower sensitivity for anti-NMDAR antibodies when FACS was used instead of visual scoring (Ramberger et al., 2015).

Over the last years the sensitivity and specificity of CBAs using serum or CSF has become increasingly clear (see Table 1). Intrathecal antibody synthesis is high in anti-NMDA, -AMPA and GABA_B_ receptor encephalitis (Lai et al., 2009; Lancaster et al., 2010; Dalmau et al., 2011), facilitating antibody detection in CSF. For anti-NMDAR antibodies, a permeabilized CBA has a sensitivity of 100% for CSF and 86% for serum (Gresa-Arribas et al., 2014). For anti-AMPAR antibodies this is 100% for CSF vs. 70% for serum (Höftberger et al., 2015), and for anti-GABA_B_R 100% for CSF and 67–93% for serum (Lancaster et al., 2010; Höftberger et al., 2013; Jeffery et al., 2013). For anti-LGI1 antibodies sensitivity on IHC is 88% and 100% for CSF and serum respectively, whereas the commercial LGI1 CBA using CSF has only 53% sensitivity, probably reflecting lower intrathecal antibody synthesis (van Sonderen et al., 2016c). Special attention is required for patients undergoing plasma exchange at the moment of serum assessment, as antibodies may no longer be detectable in serum (Florance et al., 2009).

Over the last years high throughput screenings have been published, mainly in the field of psychiatry, in which the presence of anti-neuronal surface antibodies is tested using CBA with serum only. These studies have detected anti-NR1 antibodies in up to 10% of neuropsychiatric disorders, but also in healthy individuals (Zandi et al., 2011; Dahm et al., 2014; Hammer et al., 2014). However, these studies were confusing due to lumping of IgG with IgA/IgM antibody isotypes, incomplete testing or selection bias. Only IgG subclass antibodies directed at NR1 alone causes the disease described as anti-NMDAR encephalitis. The clinical relevance of IgM and IgA antibodies is so far unclear, as was also concluded from other articles studying psychiatric populations (Steiner et al., 2013). Anti-NMDAR encephalitis can present as an isolated first psychotic episode (Kayser et al., 2013), although the presence of anti-NMDAR encephalitis among first episode psychosis patients is likely to be (less than) 1%. This chance quickly increases if patients develop neurological symptoms or additional features such as fever or autonomic dysfunction. In studies testing for anti-NMDAR IgG in a purely psychiatric population using more than one method (CBA combined with IHC or ICC of living hippocampal neurons, or the combination of serum and CSF) no patients with only psychiatric features and anti-NMDAR encephalitis have been found (Masdeu et al., 2012; van Mierlo et al., 2015). The low* a priori* chance in patients with psychiatric disorders combined with a specificity of 97–99.4% for CBA of serum (Gresa-Arribas et al., 2014) results in a *post priori* chance of only 25–60% when testing serum only. These results indicate that high throughput screening studies in a population with low disease prevalence requires excellent specificity to be of value. Combining CBA with IHC or live neuron staining could for example increase the specificity (Gresa-Arribas et al., 2014).

## Immunoprecipitation and Mass Spectrometry Analysis of Membrane Antigens

Some patients have a clinical phenotype strongly suggesting an autoimmune etiology but test negative for all known surface antigens. If IHC and live ICC provide a strong indication for the presence of cell-surface antibodies one can try to identify the molecular target of the antibodies in order to develop a CBA. Classically, novel PNS antigens were identified using cDNA expression libraries by phage display (Hufton et al., 1999). Strikingly, only intracellular antigens with mostly linear epitopes have been identified using this technique, indicating that it is less suitable for conformational epitopes. Currently, most novel surface antigens are identified by performing immunoprecipitation with patient’s serum or CSF followed by mass spectrometry analysis (IP-MS; Lai et al., 2009; Lancaster et al., 2010; de Graaff et al., 2012; Boronat et al., 2013; Petit-Pedrol et al., 2014; Sabater et al., 2014). Although in a seemingly straightforward procedure many factors are complicating the identification of a membrane antigen.

Firstly MS analysis is hampered by properties of the membrane proteins itself. They are expressed relatively low compared to cytosolic proteins. Due to their membrane spanning hydrophobic domains membrane proteins aggregate easily, leading to inefficient proteolytic cleavage. This leads to underrepresentation of membrane proteins in the sample (Helbig et al., 2010; Barrera and Robinson, 2011). Fractionation of membrane preparations, synaptosome isolation or surface biotinylation can be used to enrich (synaptic) membrane proteins in the input material. In order to reduce the detection of nonspecific cytosolic and nuclear proteins some labs perform surface labeling of primary hippocampal neurons and subsequently lyse the cells and precipitate IgG with the bound antigen (Boronat et al., 2013).

Secondly membrane proteins have the tendency to misfold when extracted by detergent. This leads to disruption of the conformational epitope and reduced antibody binding. Special attention is therefore required for the choice of detergent in order to optimize solubilization. Which detergent is suitable for membrane protein solubilization depends on the type of membrane protein that needs to be extracted (Privé, 2007). The fact that the membrane antigen is still unknown when performing IP-MS severely hampers the choice of detergent. A new amphipathic polymer that solubilizes membrane proteins in intact membrane patches might be a promising alternative (Dörr et al., 2016).

In general CSF contains less antibodies than serum, both in number and variety, and in case of intrathecal synthesis, the relative amount of specific antibodies is higher. Therefore the use of CSF in staining and immunoprecipitation is thought to provide cleaner results. However, CSF is usually less readily available than serum.

## Conclusion and Recommendations

The detection of synaptic cell-surface antibodies has significant consequences for the treatment and follow-up of AIE patients. It can confirm the autoimmune-mediated nature of the syndrome and can provide a clue for a possible underlying tumor. In order to successfully identify antibodies to surface antigens clinical assessment and patient selection by an experienced clinician is of key importance. For diagnostic purposes both serum and CSF should be tested by a combination of IHC and CBA to provide highest sensitivity and specificity. Live staining of cultured hippocampal neurons is labor intensive. The neurons used for diagnostic testing cannot be prepared beforehand and cannot be stored. However, live ICC of neurons can provide valuable additional information when the results from IHC and CBA are inconclusive.

Samples selected for IP-MS need to show robust results on both IHC and live ICC of hippocampal neurons. Preferentially patients with a similar staining pattern and clinical phenotype are grouped. If possible, two different serum samples from one patient can be used to perform IP-MS. Comparing two lists of one patient (or the lists of patients with a similar clinical phenotype and/or similar staining pattern on IHC) could facilitate the detection of novel antigens.

In the next years novel neuronal surface antigens will be identified, most likely by screening cohorts of patients with for example epilepsy or dementia. It is expected that these yet unknown patients will phenotypically show less encephalitis and more encephalopathy. As screening becomes more extensive, careful evaluation of specificity and pathogenicity of novel antibodies will be necessary. Given the low frequency of occurrence of most of these antibodies, meaningful clinical studies will require international collaboration.

## Author Contributions

MHvC-H wrote the manuscript, MJT, EdG, MWJS, PAESS and CCH have provided critical comments to the manuscript.

## Funding

This research was funded by NUTS-OHRA (1104-034), Hersenstichting (2012(1)-141).

## Conflict of Interest Statement

MHvC-H has nothing to disclose. The work of MJT is supported by grants from the Netherlands Organisation for Scientific Research (NWO, Veni-incentive and Memorabel fellowship), the Dutch Epilepsy Foundations (NEF, project 14-19) and ErasmusMC fellowship. MJT received research funds for serving on a scientific advisory board of MedImmune LLC and a travel grant for lecturing in India from Sun Pharma, India. MWJS has received financial compensation for seminars and conference visits from Biognost, Inova and Thermo Scientific. EdG and PAESS received a research grant from Euroimmun for a patent for the use of DNER as an autoantibody test. CCH has nothing to disclose.
